# The effect of angiotensin converting enzyme gene insertion/deletion polymorphism on anthropometric and biochemical parameters among hypertension patients: A case-control study from Northwest Ethiopia

**DOI:** 10.1371/journal.pone.0285618

**Published:** 2023-05-18

**Authors:** Tsegaye Adane Birhan, Meseret Derbew Molla, Kibur Hunie Tesfa

**Affiliations:** 1 Department of Environmental and Occupational Health and Safety, Institute of Public Health, College of Medicine and Health Sciences, University of Gondar, Gondar, Ethiopia; 2 Department of Biochemistry, School of Medicine, College of Medicine and Health Sciences, University of Gondar, Gondar, Ethiopia; Temple University School of Medicine, UNITED STATES

## Abstract

**Introduction:**

The angiotensin-converting enzyme (ACE) gene polymorphism has recently been linked with altered anthropometric and biochemical parameters in hypertensive patients. However, these links are still poorly understood and there is scarce evidence on the topic. Therefore, this study aimed to assess the effect of ACE gene insertion/deletion (I/D) polymorphism on anthropometric and biochemical parameters among essential hypertension patients at the University of Gondar Comprehensive Specialized Hospital, Northwest Ethiopia.

**Materials and methods:**

A case-control study with 64 cases and 64 controls was conducted from October 07, 2020, to June 02, 2021. The anthropometric measurements, biochemical parameters, and ACE gene polymorphism were determined using standard operating procedures, enzymatic colorimetric method, and polymerase chain reaction, respectively. A one-way analysis of variance was used to determine the association of genotypes with other study variables. P value < 0.05 was regarded as statistically significant.

**Result:**

The systolic/diastolic blood pressure and blood glucose level (P-value<0.05) were significantly higher among study hypertensive patients with the DD genotype. However, anthropometric measures and lipid profiles of cases and controls were not associated with ACE gene polymorphism (P-value>0.05).

**Conclusion:**

The DD genotype of the ACE gene polymorphism was found to have a significant association with high blood pressure and blood glucose levels in the study population. Advanced studies with a considerable sample size may be needed to utilize the ACE genotype as a biomarker for the early detection of hypertension-related complications.

## 1. Introduction

A systolic blood pressure (SBP) of 130 mmHg or higher and/or a diastolic blood pressure (DBP) of 80 mmHg or higher are considered to be markers for hypertension [1]. An enormous global problem, hypertension is a significant cardiovascular risk factor linked with deadly consequences [2, 3]. Additionally, it frequently co-occurs with other chronic, non-communicable diseases that can lead to early death and disability, including myocardial infarction, congestive heart failure, stroke, and chronic kidney disease [3–5].

The Rennin-angiotensin-aldosterone system (RAAS) genetic polymorphism, namely the angiotensin-converting enzyme (ACE) insertion/deletion (I/D) polymorphism, has been identified as a cardiovascular risk factor in several ethnicities [6–11]. In prior research, we found that the DD genotype of the ACE gene was related to essential hypertension in the Ethiopian population [12]. This could happen because people with the DD genotype of the ACE gene may have higher levels of plasma ACE [4, 13], which is responsible for increased synthesis of angiotensin-II, a powerful vasoconstrictor, and inactivation of bradykinin, a powerful vasodilator. This could result in increased peripheral resistance and higher blood pressure [9, 10].

Numerous epidemiological studies have found direct links between anthropometric measurements and hypertension risk [2–5, 14, 15]. Although these relationships have been regularly observed, they are still poorly understood, and the mechanical reasons for the phenomena are still being contested, with no biological model of the process produced [16]. The study conducted in Tunisia has reported a substantial association between ACE gene polymorphism and body mass index (BMI) of hypertensive individuals [17]. However, studies conducted in Brazil [18] and Pakistan [19] did not discover an association between the ACE gene I/D polymorphism and the anthropometric characteristics of hypertensive patients. However, since the ACE gene polymorphism is important for RASS pathway regulation, it is thought to have a substantial connection with altered anthropometric and biochemical characteristics of the population.

Previous research has also found a substantial association between dyslipidemia and hypertension [20–23]. Individuals with the DD genotype had more lipid abnormalities [10, 18]. Although some studies have revealed higher triglycerides (TG) and low-density lipoprotein-cholesterol (LDL-c) in D allele carriers [24], it is still unknown how the ACE gene polymorphism impacts lipid profile, which necessitates further research. Moreover, some studies have not been able to demonstrate an association between lipid abnormalities and ACE gene polymorphism [25]. As a result, the association of lipid abnormalities with different ACE gene variants among hypertensive patients remains inconsistent in different populations and is still poorly understood.

The other studies also revealed that individuals with the DD genotype had significantly higher blood glucose levels [10]. This implies that individuals with the ACE DD genotype have a higher risk of poor glucose metabolism and significantly higher blood glucose levels. This may perhaps contribute to the formation of Type II diabetes as a comorbidity. Additionally, there isn’t ample information on the development of dysglycemia during hypertension, despite growing interest in the topic.

Few studies have examined how ACE gene variation affects anthropometric and biochemical parameters in hypertensive patients [18, 24, 26]. As per our knowledge, this is the first investigation that looked at how the ACE gene I/D polymorphism affects anthropometric and biochemical measurements in the Ethiopian population. The discovery might lead to an ACE gene genotype that might be used as a biomarker for the likelihood of obesity, dyslipidemia, and type 2 diabetes mellitus among hypertensive patients for the early detection and prevention of hypertension-related comorbidities. Therefore, the present study aimed to evaluate the effect of ACE gene I/D polymorphism on anthropometric and biochemical factors among hypertensive patients at the University of Gondar Comprehensive Specialized Hospital, Northwest Ethiopia.

## 2. Methods and materials

### 2.1. Study design, setting, and period

A case-control study was conducted from October 07, 2020, to June 02, 2021, at the University of Gondar Comprehensive Specialized Hospital, Northwest Ethiopia. It is one of the largest tertiary teaching hospitals in the country and serves a catchment area of approximately five million people. It has a follow-up clinic for major chronic illnesses including hypertension.

### 2.2. Source and study population

All essential hypertensive patients and all healthy normotensive individuals who visit the hospital were the source population for cases and controls, respectively. All essential hypertensive patients who were newly diagnosed or on follow-up, as well as all healthy normotensive individuals who visited the hospital during the study period, were considered the study population for cases and controls, respectively. The eligibility criteria were addressed in our previously published work [12].

### 2.3. Sample size determination and sampling method

The sample size for this research was calculated using G* power version 3.1.9.4 software by selecting an independent *t-*test and the total sample size was 128 (64 cases and 64 controls) [12, 27]. Therefore, a 1:1 case-control ratio was recruited as study participants using a simple random sampling technique.

### 2.4. Variables of the study

The dependent variables were serum lipid profiles (serum total cholesterol (TC), TG, HDL-c, and LDL-C) and Fasting blood glucose (FBG) level, whereas ACE gene polymorphism was taken as an explanatory variable.

### 2.5. Blood sampling and data collection

#### 2.5.1. Data collection procedure

The socio-demographic data and clinical characteristics were collected using semi-structured interviewer-administered questionnaires and medical record reviews. The anthropometric such as body mass index (BMI) and waist-to-hip ratio (WHR) and blood pressure measurements, blood sample collection, DNA extraction, and amplification were done through the standard procedure [12].

#### 2.5.2. Blood samples collection and biochemical analysis

Five-milliliter fasting venous blood sample was collected using a serum separator test tube by following an aseptic blood collection procedure. The serum was separated from the whole blood by centrifuging at 3,000 RPM for 5 minutes. Then, the separated serum was analyzed for glucose and lipid profiles through an enzymatic colorimetric method by using a Mindray BS-200E chemistry analyzer (Shenzhen Mindray Bio-Medical Electronics Co. Ltd, China) following standard operating procedures. The interpretation of test results was based on the manufacturers’ manual for each analyte measured.

### 2.6. Data quality control and management

As mentioned in our previously published work [12], training of data collectors, pretest, and use of standard operating procedures in the pre-analytic, analytic, and post-analytic stages of laboratory services were used to assuring data quality. Repeated genotype was also done for about 10% of randomly selected samples as means of conformation.

### 2.7. Data processing and analysis

Data were analyzed using SPSS software, version 25.0 (IBM Inc.). Mean and standard deviations were used to describe the features of the study subjects. A one-way analysis of variance with the Least Significant Difference (LSD) post-hoc test was used to determine the association of genotypes with anthropometric and biochemical parameters. The variables with p-value < 0.05 were declared statistically significant.

### 2.8. Ethical statements

The Institutional Review Board of the University of Gondar granted ethical approval for this study (Ref. No: 2094/07/2020). After a brief explanation of the purpose, benefits, and risks of the study, written informed consent was obtained from the respondents. The confidentiality of the participants was assured by leaving out their names and telling the safety of the place where the data will be stored after collection.

## 3. Result

### 3.1. Socio-demographic characteristics of study participants

This study had a total of 128 participants—64 hypertension patients and 64 controls—with a 100% response rate. In our earlier published work, we reported and discussed the socio-demographic and behavioral features of the study participants. The ACE genotypes frequency distribution in hypertensive patients and healthy control is reported also in our previously published work [12].

### 3.2. Biochemical characteristics of study participants

The BMI, WHR, SBP, DBP, TG, TC, LDL-c, and FBG levels were significantly higher in hypertensive patients than in controls, while HDL-c was significantly higher in controls than in hypertensive patients (p-value < 0.05) (**Table 1**).

**Table 1:** Anthropometric and biochemical measurements of study participants (n = 128).

Characteristics	Study participants		P-value
	Cases (N = 64)	Control (N = 64)	
	Mean ±SD	Mean ±SD	
BMI (kg/m^2^)	24.94±5.19	22.29±3.46	0.001*
WHR	0.91±0.20	0.82±0.09	0.001*
SBP (mmHg)	132.50±11.20	107.19±9.34	0.001*
DBP (mmHg)	82.03±8.58	71.41±8.89	0.001*
TG (mg/dl)	133.22±43.03	105.48±41.07	0.001*
TC (mg/dl)	171.33±41.25	145.55±21.88	0.001*
HDL-c (mg/dl)	49.90±11.53	60.17±11.86	0.001*
LDL-c (mg/dl)	94.79±34.50	64.29±16.10	0.001*
FBG (mg/dl)	111.75±38.70	81.16±29.49	0.001*

BMI = Body Mass Index, WHR = Waist-to-Hip Ratio, SBP = Systolic Blood Pressure, DBP = Diastolic Blood Pressure, TG = Triglyceride, TC = Total Cholesterol, HDL-c = High Density Lipoprotein Cholesterol, LDL-c = Low Density Lipoprotein Cholesterol, FBG = Fasting Blood Glucose. *statistically significant at p-value <0.05.

### 3.3. Association of ACE gene I/D polymorphism with anthropometric and biochemical characteristics of hypertensive patients

Anthropometric measures (BMI and WHR) and lipid profiles of hypertensive patients showed no association with ACE gene polymorphism (p-value > 0.05). There were significant differences in the mean SBP, DBP, and FBG levels among hypertensive patients with different ACE genotypes (p-value =  0.003, 0.002, and 0.025, respectively) (**Table 2**).

**Table 2:** Effect of ACE I/D polymorphism on anthropometric and biochemical features of cases (n = 64).

Variables	Cases (n = 64)			p-value
	DD (n = 31)	ID (n = 19)	II (n = 14)	
BMI	24.22±5.34	24.56±3.54	27.11±6.41	0.21
WHR	0.94±0.18	0.87±0.28	0.93±0.08	0.538
SBP (mmHg)	136.61±10.68	130.53±8.32	126.43±6.02	**0.003** *
DBP (mmHg)	85.48±6.24	80.53±9.11	76.43±9.29	**0.002** *
TG (mg/dl)	134.77±38.31	128.11±41.56	136.71±56.01	0.823
TC (mg/dl)	178.46±44.74	160.25±28.83	170.59±46.63	0.322
HDL-c (mg/dl)	48.89±11.05	52.58±13.50	48.51±9.78	0.488
LDL-c (mg/dl)	102.61±37.63	82.05±24.13	94.74±36.22	0.123
FBG (mg/dl)	124.65±51.91	103.68±11.06	94.14±6.66	**0.025** *

BMI = Body Mass Index, WHR = Waist-to-Hip Ratio, SBP = Systolic Blood Pressure, DBP = Diastolic Blood Pressure, TG = Triglyceride, TC = Total Cholesterol, HDL-c = High Density Lipoprotein Cholesterol, LDL-c = Low Density Lipoprotein Cholesterol, FBG = Fasting Blood Glucose.

*. The mean difference is significant at the 0.05 level.

The LSD post-hoc test showed that SBP and DBP were significantly higher in hypertensive patients with the DD genotype as compared to those with II and ID genotypes (P-value < 0.05) (**Table 3**). This indicates the DD genotype was a significant predictor of the blood pressure and fasting blood glucose levels of the hypertensive patients.

**Table 3:** Post hoc comparison of the effect of each genotype on the blood pressure and fasting blood glucose levels of hypertensive patients (n = 64).

Variable	ACE gene polymorphism	Mean Difference (95% CI)	Sig.
SBP (mmHg)	DD vs ID	6.09 (0.74, 11.43)	**0.026** *
	DD vs II	10.18 (4.28, 16.09)	**0.001** *
	ID vs II	4.10 (-2.36, 10.56)	0.21
DBP (mmHg)	DD vs ID	4.96 (0.37, 9.55)	**0.035** *
	DD vs II	9.06 (3.98, 14.13)	**0.001** *
	ID vs II	4.10 (-1.45, 9.64)	0.145
FBG (mg/dl)	DD vs ID	20.96 (-0.61, 42.53)	0.057
	DD vs II	30.50 (6.66, 54.34)	**0.013** *
	ID vs II	9.54 (-16.54, 35.62)	0.467

SBP = Systolic Blood Pressure, DBP = Diastolic Blood Pressure, FBG = Fasting Blood Glucose, CI = Confidence Interval.

*. The mean difference is significant at the 0.05 level.

### 3.4. Association of ACE gene I/D polymorphism with anthropometric and biochemical characteristics of healthy controls

In controls, the BMI, WHR, DBP, and lipid profiles were not significantly associated with ACE gene I/D polymorphism (P-value >0.05). There were significant differences in the mean SBP and FBG levels among normotensive controls with various ACE genotypes (p-value =  0.013 and 0.005, respectively) (**Table 4**).

**Table 4:** Effect of ACE I/D polymorphism on anthropometric and biochemical features of controls (n = 64).

Variables	Controls (n = 64)			p-value
	DD (n = 19)	ID (n = 16)	II (n = 29)	
BMI	23.01±3.27	21.74±2.84	22.12±3.89	0.53
WHR	0.81±0.13	0.85±0.06	0.81±0.06	0.374
SBP (mmHg)	111.58±7.46	107.50±7.75	104.14±9.07	**0.013** *
DBP (mmHg)	74.74±6.76	71.25±8.06	69.31±7.64	0.057
TG (mg/dl)	116.00±43.51	106.31±38.02	98.14±40.89	0.342
TC (mg/dl)	143.02±21.87	152.04±18.94	143.63±23.36	0.396
HDL-c (mg/dl)	59.21±10.39	62.73±10.62	59.38±13.48	0.614
LDL-c (mg/dl)	60.61±15.08	68.04±17.12	64.62±16.21	0.398
FBG (mg/dl)	97.89±48.90	81.38±10.00	70.07±6.98	**0.005** *

BMI = Body Mass Index, WHR = Waist-to-Hip Ratio, SBP = Systolic Blood Pressure, DBP = Diastolic Blood Pressure, TG = Triglyceride, TC = Total Cholesterol, HDL-c = High Density Lipoprotein Cholesterol, LDL-c = Low Density Lipoprotein Cholesterol, FBG = Fasting Blood Glucose.

*. The mean difference is significant at the 0.05 level.

Based on the LSD post hoc result, the SBP and FBG levels were significantly higher among controls with the DD genotype than the controls with the II genotype (p-value = 0.003 and 0.001, respectively) (**Table 5**). Similarly, as in the case of hypertensive patients, this result implies DD genotype was a significant predictor of the blood pressure and fasting blood glucose levels of the controls.

**Table 5:** Post hoc comparison of the effect of each genotype on the systolic blood pressure and fasting blood glucose levels of controls (n = 64).

Variable	ACE gene polymorphism	Mean Difference (95% CI)	Sig.
SBP (mmHg)	DD vs ID	3.58 (-2.13, 9.28)	0.214
	DD vs II	7.58 (2.74, 12.42)	**0.003** *
	ID vs II	4.00 (-1.22, 9.22)	0.131
FBG (mg/dl)	DD vs ID	16.56 (-2.41, 35.54)	0.086
	DD vs II	27.43 (11.32, 43.53)	**0.001** *
	ID vs II	10.87 (-6.50, 28.24)	0.216

SBP = Systolic Blood Pressure, FBG = Fasting Blood Glucose, CI = Confidence Interval

*. The mean difference is significant at the 0.05 level.

## 4. Discussion

In our previous work [12], the ACE gene polymorphism was investigated in Ethiopian hypertensive patients and the corresponding healthy controls. We found that the DD genotype and D allele of the ACE gene predispose to the occurrence of hypertension in our study participants. However, the influence of the ACE gene polymorphism on the anthropometric and biochemical parameters of hypertension patients remains unresolved. Therefore, the current study is the first one to be done in the Ethiopian population and assessed the effect of the ACE genotype on the anthropometric measures and biochemical features of essential hypertension.

### 4.1 Anthropometric and biochemical parameters among study subjects

The BMI, WHR, SBP, DBP, TG, TC, LDL-c, and FBG levels were significantly higher in hypertensive patients than in controls while HDL-c was significantly higher in controls than hypertensive patients (p-value <0.05).

### 4.2 Effect of ACE gene polymorphism on anthropometric parameters

The present study found that hypertensive patients had significantly higher BMI and WHR than normal controls. It could be a result of the linkage between obesity and higher blood pressure which may cause obesity-induced hypertension through the mechanism of insulin resistance, sodium retention, increased sympathetic nervous system activity, activation of renin-angiotensin-aldosterone, and altered vascular function [28, 29]. This finding was in line with the findings from Bangladesh [20], China [30], and South Korea [31], while it was inconsistent with the finding of studies conducted in southern India [10] and Egypt [32]. The inconsistency might be due to the variation in the ethnicity and socio-demographic condition of the study subjects. The anthropometric measurements (BMI and WHR), both in the case and control groups, did not substantially associate with the ACE genotypes. This finding is in line with the earlier findings [33, 34].

### 4.3 Effect of ACE gene polymorphism on blood pressure

The outcomes of the current investigation revealed that hypertensive patients with the DD genotype had considerably higher SBP and DBP than those with the ID and II ACE genotypes. Similarly, controls with the DD genotype ACE gene had significantly higher SBP than those with the II genotype. This could be justified by the possibility that people with the DD genotype may have greater plasma ACE levels, which are responsible for enhanced angiotensin-II synthesis and inactivation of bradykinin, a potent vasodilator. This might affect the muscle of the arteries, heighten peripheral resistance, and raise blood pressure [9, 10]. High levels of angiotensin-II can also trigger the adrenal glands to produce aldosterone, which in turn stimulates the kidneys’ epithelial cells to promote salt and water absorption, leading to an increase in blood volume and blood pressure [13, 32].

### 4.4 Effect of ACE gene polymorphism on lipid profile

Triglyceride, total cholesterol, and LDL-c were also found to be significantly higher in hypertension patients than in normotensive controls. This outcome is in line with the other studies conducted in southern India [10], Burkina Faso [35], north India [25], and South Korea [31]. It was inconsistent with a study reported from China [8] which found no significant association between TC, TG, and HDL-c. The study conducted in Egypt [36] also found the only significant increase in triglyceride levels in hypertensive cases.

On the other hand, some studies also documented that there was no significant difference in the mean TC values between the patients and control groups in Korea [31, 37]. These variations might be due to sample size differences and study subject variation in race. The HDL-c was also found to be significantly lower in cases than in the controls. The finding was consistent with a similar study reported from South Korea [31], Southern India [10], and the Himachal Pradesh and Punjab areas of India [37]. Additionally, it was incongruent with the findings of the Egyptian study, which showed that hypertensive patients had greater levels of HDL-c [36]. Prior studies also found an abnormality in the lipid profile of hypertensive individuals [20].

One possible explanation for these relationships is that hypertension and dyslipidemia share common pathophysiological etiologies, such as obesity and the resulting deregulated release of adipocytokine from adipose tissue [22]. Moreover, dyslipidemia also hurts the anatomical and functional characteristics of the arteries by forming cholesterol deposits on blood vessel walls and forming hard plaque through precipitating calcium ions. This leads to the hardening and narrowing of the arteries with cholesterol plaque and calcium (atherosclerosis), and the heart has to strain much harder to pump blood through them. As a result, blood pressure becomes abnormally high [16, 21].

Conversely, higher blood pressure among hypertensive cases would result in increased renal tubular reabsorption and increased arterial pressure. This would also lead to higher values of glomerular capillary wall stress, activation of neuro-humoral systems, and increased serum lipid level among cases [38]. This implies that high TG, high LDL-c, and low HDL-c levels are associated with increased blood pressure and atherosclerosis [16]. This also implies that hypertension can coexist with other risk factors, especially dyslipidemia, which may act synergistically in the pathogenesis of atherosclerosis disease.

When comparing the lipid profiles across various ACE genotypes, none of the lipid profiles (TC, TG, LDL-c, and HDL-c) were significantly associated with the ACE gene I/D polymorphism both in cases and controls. This finding similarly supported the earlier findings [33, 34]. Contrarily, it contradicts studies that found a significant association between ACE gene polymorphism and lipid abnormalities [39, 40].

### 4.5 Effect of ACE gene polymorphism on fasting blood glucose

The blood glucose level was also found to be significantly higher among hypertensive cases than the healthy controls. This could be because elevated blood pressure alters the supply of insulin and glucose to skeletal muscle cells, resulting in decreased glucose absorption and increased plasma glucose levels [41]. In addition, hypertension may induce microvascular dysfunction through an increase in the production of superoxide anions and hydrogen peroxide (levels of reactive oxygen species) and a decrease in nitric oxide synthesis [42, 43] which may result in insulin resistance and hyperglycemia. Previous studies also documented that biomarkers of endothelial dysfunction were found to be independent predictors of hyperglycemia [38]. This implies hypertension would contribute to the pathophysiology of type II diabetes development. This finding was in line with the findings in previous studies conducted in Debre Markos, Ethiopia [4], southern Ethiopia [38], southern India [10], and northern India [25]. The finding was also inconsistent with another study conducted in Egypt [32].

When comparing the FBG level across various ACE genotypes, the present research found that hypertensive patients and controls with the DD genotype had considerably higher mean FBG levels than their corresponding individuals with the II genotype. This may be described by the greater plasma ACE levels among DD genotype carriers, which led to increased angiotensin-II levels [9, 10], which could potentially prevent the activation of the insulin-mediated phosphoinositide 3-kinase (PI3K) signaling cascade, affecting endothelial nitric oxide generation and glucose transporter 4 (Glut-4) translocation in insulin-sensitive tissues, resulting in vascular and systemic insulin resistance, respectively [36, 44]. This implies people with the DD genotype appear to have an increased risk of poor glucose metabolism and significantly higher blood glucose levels. Furthermore, earlier research has established that ACE inhibitors may enhance glucose utilization and reduce hepatic glucose synthesis [36]. This could be because the D allele has been linked to lower insulin secretion during the first half hour of an oral glucose tolerance test [45], while the I allele has been linked to a higher percentage of slow-twitch type I fibers in human skeletal muscle [46], which could affect glucose metabolism.

In summary, the current investigation discovered that the ACE gene I/D polymorphism is linked to the abnormal blood pressure (both SBP and DBP) and blood glucose levels of study participants. The result of this study supports the hypothesis that the DD genotype has a strong association with increased blood pressure and altered blood glucose level that implicates the ACE polymorphism plays an important role in the development of Type 2 diabetes.

### 4.6 Strengths and limitations

As a strength, this study is the first study that was conducted to evaluate ACE gene polymorphism and its effect on the anthropometric and biochemical factors in Ethiopian hypertensive cases and their respective healthy controls. Furthermore, selection bias was minimized by taking matched cases and controls in terms of sex and age variable, which are non-modifiable risk factors.

The main limitation of our study was the limited sample size. Although the assay for serum ACE could not be performed, such tests might have provided a more accurate assessment of the relationship between ACE genotype and biochemical characteristics.

## 5. Conclusion

In conclusion, higher blood pressure and blood glucose levels were strongly related to the DD genotype of the ACE gene. The ACE gene I/D polymorphism could therefore be employed as a biomarker for the early detection of hypertension consequences such as Type II diabetes and metabolic syndrome. To clarify the relationship between the ACE gene I/D polymorphism and altered anthropometric and biochemical parameters, additional studies with substantial sample sizes of individuals from various ethnic backgrounds and plasma ACE measurements are imperative.

## Supporting information

S1 File Standard operating procedures for biochemical analysis. (DOCX)

S1 Data (XLSX)
