# Supplementary material for: The effect of angiotensin converting enzyme gene insertion/deletion polymorphism on anthropometric and biochemical parameters among hypertension patients: A case-control study from Northwest Ethiopia
Source: PLoS One. 2023 May 18;18(5):e0285618. doi: 10.1371/journal.pone.0285618 (PMC10194856; doi:10.1371/journal.pone.0285618)
Supplement: S1 File — (DOCX) [file pone.0285618.s001.docx]

**Standard Operating Procedures for Biochemical Analysis**

Five milliliter fasting venous blood sample was collected using serum separator test tube by following aseptic blood collection procedure. Serum was separated from the whole blood by centrifuging at 3,000 revolutions per minute for 5 minutes. Then, the separated serum was analyzed for glucose and lipid profiles by enzymatic colorometric method using Mindray BS-200E chemistry analyzer (Shenzhen Mindray Bio-Medical electronics Co. Ltd, China). The interpretation of test results were based on the manufacturers’ manual for each analyte measured.

**Blood Glucose level**

*Test principle:*

Glucose level was determined by an enzymatic spectrophotometric glucose oxidase method. The basic principle is that, Glucose is oxidized by glucose oxidase (GOD) enzyme to produce gluconate and hydrogen peroxide (H_2_O_2_). The H_2_O_2_ is then oxidatively coupled with 4 amino-antipyrene (4-AAP) and phenol in the presence of peroxidase (POD) enzyme to yield a red Quinoneimine quinoeimine dye that is measured at 505 nm with a spectrophotometer. The absorbance at 505 nm is proportional to concentration of glucose in the sample. The method has linearity from 0.0126 mmol/l (0.23 mg/dl) to 27.5 mmol/l (500 mg/dl).

Glucose +2H_2_O + O_2_ GOD Gluconate + H_2_O_2_

2H_2_O_2_+ 4-AAP+ Phenol POD Quinoeimine Dye (pink chromogen) + 4H_2_O

Absorbance of the colored solution is directly proportional to the glucose concentration when measured at 505 nm.

**Triglyceride**

*Test principle:*

Enzymatic colorimetric method (GPO/PAP) with glycerol phosphate oxidase and 4 amino phenazone

Triglycerides are hydrolyzed by lipoprotein lipase (LPL) to glycerol and fatty acids. Glycerol is then phosphorylated to glycerol-3-phosphate by ATP in a reaction catalyzed by glycerol kinase (GK). The oxidation of glycerol-3-phosphate is catalyzed by glycerol phosphate oxidase (GPO) to form dihydroacetone phosphate and hydrogen peroxide (H2O2). In the presence of peroxidase (POD), hydrogen peroxide affects the oxidative coupling of 4-chlorophenol and 4-aminophenazone to form a red colored quinoneimine dye, which is measured at 512 nm. The increase in absorbance is directly proportional to the concentration of triglycerides in the sample. (65).

Triglycerides + 3H_2_O  *lipase* glycerol + fatty acids

Glycerol + ATP *glycerokinase* glycerol-3-phosphate + ADP

Glycerol-3-phosphate + O_2_ *glycerophosphate oxidase* Dihydroxyacetone phosphate + H_2_O_2_

2H_2_O_2_ + 4-aminophenazone + 4-chlorophenol *peroxidase* Quinoneimine dye + 4-chlorophenol+4H_2_O

Desirable or normal fasting triglyceride levels were considered to be those below 150 mg/dL, and increased range 150 mg/dL or above.

**Cholesterol**

*Test principle:*

Enzymatic colorimetric method (CHOD/PAP) with cholesterol esterase, cholesterol oxidase, and 4-aminoantipyrine

Cholesterol esterase (CE) hydrolyzes cholesterol esters to form free cholesterol and free fatty acids. Cholesterol oxidase (CHOD) then catalyzes the oxidation of cholesterol to form cholest-4-ene-3-one and H_2_O_2_. In the presence of peroxidase (POD), the hydrogen peroxide formed affects the oxidative coupling of phenol and 4-amino-antipyrine (4-AAP) to forma red colored Quinoneimine dye. The color intensity of the red Quinoneimine dye formed is directly proportional to the cholesterol concentration. It is determined by measuring the increase in absorbance at 520 nm.

Cholesterol ester + H_2_O *cholesterol ester hydrolase* cholesterol + fatty acid

Cholesterol + O_2_ *cholesterol oxidase* cholest- 4-en-3-one + H_2_O_2_

2H_2_O_2_+4-aminophenazone + phenol *phenol peroxidase* 4-(p-benzoquinone-monoimino) (Quinoneimine dye) + 4 H_2_O

Desirable or normal cholesterol levels were considered to be those below 150 mg/dL, and it was considered abnormally elevated levels of cholesterol to be those 150 mg/dL or above.

**HDL-Cholesterol:**

*Test principle:*

Homogeneous enzymatic colorimetric assay

In the presence of magnesium sulfate and dextran sulfate, water-soluble complexes with LDL, VLDL, and chylomicrons are formed which are resistant to Poly Ethylene Glycol (PEG)-modified enzymes. The cholesterol concentration of HDL-cholesterol is determined enzymatically by cholesterol esterase and cholesterol oxidase coupled with PEG to the amino groups (approximately 40%). Cholesterol esters are broken down quantitatively into free cholesterol and fatty acids by cholesterol esterase. In the presence of oxygen, cholesterol is oxidized by cholesterol oxidase to 4-cholestenone and hydrogen peroxide. The color intensity of the blue quinoneimine dye formed is directly proportional to the HDL-cholesterol concentration. It is determined by measuring the increase in absorbance at 583 nm.

HDL-cholesteryl esters *PEG-cholesteryl esterase* HDL-unesterified cholesterol + Fatty acid.

Unesterified chol + O_2_ *PEG-cholesterol oxidase* 4 - cholestenone + H2O2.

H_2_O_2_ + 5-aminophenazone + N-ethyl-N-(3-methylphenyl)-N’succinyl ethylene diamine + H_2_O + H+  *peroxidase* qunoneimine dye (purple blue pigment) + H_2_O.

A low HDL-cholesterol concentration was considered to be a value below 40 mg/dL for male and below 50 mg/dL for female. HDL cholesterol value was also used in the calculation of LDL-cholesterol.

**LDL-Cholesterol:**

*Test principle:*

Homogeneous enzymatic colorimetric assay

This automated method for the direct determination of LDL-cholesterol takes advantage of the selective micellarysolubilization of LDL-cholesterol by a nonionic detergent and the interaction of a sugar compound and lipoproteins (VLDL and chylomicrons). When a detergent is included in the enzymatic method for cholesterol determination (cholesterol esterase and cholesterol oxidase coupling reaction), the relative reactivities of cholesterol in the lipoprotein fractions increase in this order: HDL < chylomicrons < VLDL < LDL. In the presence of Mg++, a sugar compound markedly reduces the enzymatic reaction of the cholesterol measurement in VLDL and chylomicrons. The combination of a sugar compound with detergent enables the selective determination of LDL-cholesterol in the serum. In the presence of oxygen, cholesterol is oxidized by cholesterol oxidase to 4-cholestenone and hydrogen peroxide. The color intensity of the blue quinoneimine dye formed is directly proportional to the LDL-cholesterol concentration. It is determined by measuring the increase in absorbance at 583 nm.

LDL-cholesterol ester + H_2_O⎯detergent *cholesterolesterase* cholesterol +free fatty acid (selective micellary solubilization)

LDL-cholesterol + O_2_ *cholesterol oxidase* 4-cholestenone+ H_2_O_2_

2 H_2_O_2_ + 4-aminoantipyrine + HSDA+ H+H_2_O_2_ *peroxidase* purple blue pigment +5 H_2_O

Desirable level of LDL-cholesterol was that below130 mg/dL in adults; and 130mg/dL or above was considered to be elevated.
